# A Syngeneic Mouse B-Cell Lymphoma Model for Pre-Clinical Evaluation of CD19 CAR T Cells

**DOI:** 10.3791/58492

**Published:** 2018-10-16

**Authors:** Gray Kueberuwa, Weiming Zheng, Milena Kalaitsidou, David E. Gilham, Robert E. Hawkins

**Affiliations:** ^1^Manchester Cancer Research Centre Building, Department Cancer Sciences, University of Manchester; ^2^Celyad

**Keywords:** Cancer Research, Issue 140, CAR T cells, TRUCKs, adoptive T-cell therapy, syngeneic mouse model, A20, immunotherapy, IL-12, pre-conditioning, CD19, lymphoma

## Abstract

The astonishing clinical success of CD19 chimeric antigen receptor (CAR) T-cell therapy has led to the approval of two second generation chimeric antigen receptors (CARs) for acute lymphoblastic leukemia (ALL) andnon-Hodgkin lymphoma (NHL). The focus of the field is now on emulating these successes in other hematological malignancies where less impressive complete response rates are observed. Further engineering of CAR T cells or co-administration of other treatment modalities may successfully overcome obstacles to successful therapy in other cancer settings.

We therefore present a model in which others can conduct pre-clinical testing of CD19 CAR T cells. Results in this well tested B-cell lymphoma model are likely to be informative CAR T-cell therapy in general.

This protocol allows the reproducible production of mouse CAR T cells through calcium phosphate transfection of Plat-E producer cells with MP71 retroviral constructs and pCL-Eco packaging plasmid followed by collection of secreted retroviral particles and transduction using recombinant human fibronectin fragment and centrifugation. Validation of retroviral transduction, and confirmation of the ability of CAR T cells to kill target lymphoma cells *ex vivo*, through the use of flow cytometry, luminometry and enzyme-linked immunosorbent assay (ELISA), is also described.

Protocols for testing CAR T cells *in vivo* in lymphoreplete and lymphodepleted syngeneic mice, bearing established, systemic lymphoma are described. Anti-cancer activity is monitored by *in vivo* bioluminescence and disease progression. We show typical results of eradication of established B-cell lymphoma when utilizing 1^st^ or 2^nd^ generation CARs in combination with lymphodepleting pre-conditioning and a minority of mice achieving long term remissions when utilizing CAR T cells expressing IL-12 in lymphoreplete mice.

These protocols can be used to evaluate CD19 CAR T cells with different additional modification, combinations of CAR T cells and other therapeutic agents or adapted for the use of CAR T cells against different target antigens.

**Figure:**
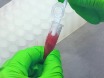


## Introduction

Chimeric antigen receptor (CAR) T-cell therapy has shown astonishing clinical success in the treatment of CD19^+^ malignancies leading to the approval of tisagenlecleucel for relapsed acute lymphoblastic leukaemia^1^ and axicabtagene ciloleucel for progressive large B-cell non-Hodgkin lymphoma^2^ in 2017.

The importance of Interactions between cancer and the immune system in both disease progression and therapeutic mechanisms is becoming increasingly recognized^3^,^4^,^5^. For example, it is well documented that the tumor microenvironment (TME) is awash with factors that can suppress the effector functions of immune cells^6^,^7^,^8^. Alternatively priming of endogenous immune cells and epitope spreading can be key in tumor eradication and long term resistance to tumor challenge^9^,^10^. Both of these phenomena cannot be evaluated in xenogeneic models that lack an immune system. Likewise, systems utilizing transgenic proteins do not accurately reflect the challenge of breaking immune tolerance which is required for epitope spreading^11^,^12^. A syngeneic model with a fully functional immune system is, therefore, paramount for modeling these important aspects of cancer disease progression and immune therapeutics.

An important caveat of CAR T-cell therapy is that lymphodepleting pre-conditioning is required for therapeutic success^13^,^14^. This is typically achieved in patients by administering chemotherapy prior to infusion of CAR T cells^15^,^16^. As a standard method, in order to mimic lymphodepletion used in the patient setting, we administer 5 Gy total body irradiation (TBI) to achieve lymphodepletion prior to administration of therapeutic CAR T cells to mice bearing systemic A20 B-cell lymphoma.

While lymphodepleting pre-conditioning is not an issue for the majority of patients, toxicity that comes with chemotherapeutic agents means that patients of low performance status are not eligible for CAR T-cell therapy. To create a test system that represents the patients ineligible for lymphodepletion, we established a lymphoreplete syngeneic mouse model in which we model CAR T-cell therapy of lymphoma. In this model, we showed that the secretion of IL-12 from within CAR T cells could lead to eradication of established lymphoma with a success rate of ~ 25%^17^. Moreover, we showed that endogenous immune cells were involved in cancer eradication.

Here we describe in detail the protocol for the production of mouse CAR T cells, establishing lymphoma in syngeneic mice, and treatment of lymphoma with CAR T cells with or without the use of lymphodepleting pre-conditioning. This can be used for combination studies of CAR T cells with other agents, testing CAR T cells with other transgenes or for the use of other adoptive cell therapy or immunotherapy strategies against lymphoma.

## Protocol

All animal experiments were conducted under the auspices of the Animals (Scientific Procedures) Act 1986 and under UK Coordinating Committee for Cancer Research guidelines. All animal studies were conducted at the CRUK-Manchester institute and approved by the local animal welfare and ethics review body (CRUK-MI AWERB).

### 1. Preparations

1. Maxiprep pMP71 retroviral construct plasmid and pCL-Eco retrovirus packaging plasmids^18^. **NOTE:** pMP71 encodes mCherry and the CAR separated by an FMDV2A sequence. This is interchangeable with other retroviral constructs. pCL-Eco encodes gag, pol and the ecotropic envelope proteins.
2. Prepare complete T cell medium (TCM) for culturing mouse T cells using RPMI 1640 medium, 10% FCS, 1% 100x penicillin-streptomycin-glutamine (PSG). **NOTE:** The solution contains 100 IU/mL penicillin, 100 µg/mL of streptomycin and 2 mM of L-glutamine), 50 μM β-mercaptoethanol and 25 mM 4-(2-hydroxyethyl)-1-piperazineethanesulfonic acid (HEPES).
3. Culture A20 cells in RPMI 1640, 10% FCS and 0.05 mM β-mercaptoethanol at 37 °C, 5% CO_2_.
4. Culture the Platinum-E (Plat-E) cells in complete Dulbecco's modified eagle medium (DMEM) (DMEM with 10% fetal calf serum (FCS), 2 mM L-glutamine, 1 μg/mL puromycin and 10 μg/mL blasticidin) at 37 °C, 5% CO_2._ **NOTE: **Plat-E cells are derived from 293T cells and express gag, pol and ecotropic envelope retroviral proteins.
5. Prepare transfection media solutions 1 and 2 immediately prior to transfection. Prepare solution 1 (pH 7.9) to contain DMEM + 10% FCS + 25 mM HEPES, solution 2 (pH 7.1) to contain DMEM + 25 mM HEPES.
6. Prepare 10 μg/mL recombinant human fibronectin fragment solution by diluting with sterile phosphate-buffered saline (PBS) and store at -20 °C until use.
7. Sterile filter all media through 0.2 μm filters prior to use (excluding recombinant human fibronectin fragment).

### 2. Retroviral Transduction of T cells

1. **Day 1: Preparation for transfection**
  1. Seed 7.5 x 10^6 ^Platinum-E (Plat-E) cells in 15 cm^2^ tissue culture dishes in 18 mL of complete DMEM and incubate overnight at 37 °C, 5% CO_2_.
2. **Day 2: Transfection of Plat-E retroviral packaging cell line**
  1. Prepare 20.4 μg of pcl-Eco packaging vector DNA, 39.6 μg of plasmid DNA encoding retroviral CAR construct and 150 μL of 1 M CaCl_2_ to final volume of 3 mL transfection solution 2 per 15 cm^2^ dish to be transfected. Vortex for 10 s and rest for 5 min
  2. Remove DMEM media from the 15 cm^2^ dishes and replace with 12 mL of transfection solution 1. **CAUTION **When changing the media, the 15 cm^2^ dishes can dry at the center. This can cause substantial death of transfected Plat-E cells. Work swiftly and remove media from just 1-2 plates at a time.
  3. Add 3 mL transfection solution 2 containing DNA and CaCl_2_ to each 15 cm^2^ dish drop-wise, evenly across each plate. Gently rock plates with a side to side motion for 10 s. Incubate at 37 °C, 5% CO_2_ overnight.
3. **Day 3: Preparation of virus-containing supernatant for transduction**
  1. Replace the media of transfected Plate-E cells with 18 mL complete TCM and return to incubator. **CAUTION **When changing the media 15 cm^2^ dishes can dry at the center. This can cause substantial death of transfected Plat-E cells. Work swiftly and remove media from just 1-2 plates at a time.
4. **Day 3: Isolation and *in vitro *activation of mouse splenic T cells**
  1. Remove spleens from 6-8-week-old BALB/c mice as previously described by Parkinson* et al.*^19^ and immerse them in sterile, ice-cold, PBS in a 50 mL conical tube.
  2. Use tweezers to transfer a spleen to a 1.5 mL microcentrifuge tube and homogenize using a pestle with minimal force.
  3. Use a 1000 μL pipette and ~ 800 μL PBS to transfer homogenate to a 100 μm pore cell strainer affixed to a 50 mL tube containing 5 mL PBS to attain a single cell suspension. Repeat step 2.4.2 for the additional spleens. Do not exceed 3 spleens per tube. **CAUTION** Splenocytes passed through filter can form clumps if left standing. Manually swirl tubes intermittently if processing several spleens to avoid cell clumping. Remaining fragments on the cell strainer can be further mashed using a plunger from a 5 mL syringe using minimal force.
  4. Top up to 20 mL with PBS. Layer the 20 mL cell suspension gently onto 20 mL of density gradient media (**Table of Materials**) in a 50 mL tube. Centrifuge the resultant overlaid suspension at 800 x g for 20 min with no brake applied.
  5. Harvest cells at interface layer using a sterile Pasteur pipette and transfer to a 50 mL tube. Top up to 50 mL with PBS and centrifuge at 800 x g for 10 min to wash. Discard the supernatant and re-suspend cells in complete TCM.
  6. Count the number of cells using a hemocytometer.
  7. Culture cells at a density of 5 x 10^6^ cells/mL in complete TCM with 30 ng/mL anti-CD3ε antibody (Clone 145-2C11), 30 ng/mL anti-CD28 antibody (Clone 37.51), 100 U/mL recombinant human IL-2 and 2 ng/mL recombinant murine IL-7. Use an appropriately sized tissue culture flask for the volume of cells harvested. **NOTE: **Antigen**-**presenting cells are required for T-cell activation by CD3 and CD28 antibodies, if working with purified T cells it is necessary to coat plates with antibodies, or use magnetic beads (**Table of Materials**)
  8. Incubate mouse splenocytes at 37 °C, 5% CO_2_ overnight.
5. **Day 3: Preparation of plates for transduction**
  1. Coat non-tissue-culture 6-well plates with 2 mL of 10 μg/mL recombinant human fibronectin fragment and incubate overnight at 4 °C.
6. **Day 4: Transduction of mouse T cells**
  1. Transfer recombinant human fibronectin fragment from coated plates to fresh non-tissue-culture 6-well plates. Incubate these plates overnight at 4 °C for round 2 of transduction.
  2. Add 2 mL of TCM to each well of original recombinant human fibronectin fragment-coated plates and leave for 30 min at room temperature to block non-specific binding.
  3. Harvest retrovirus-containing supernatant from transfected Plat-E cells in 15 cm tissue culture dishes and replace with 18 mL of complete TCM. **CAUTION **Work swiftly to avoid drying of Plat-E cells. **NOTE:** Success of transfection can be checked at this stage by fluorescence microscopy if utilizing a fluorescent marker gene such as mCherry (Figure 1).
  4. Filter the retrovirus-containing supernatant through 0.45 μm filter to remove cell debris. Remove TCM from recombinant human fibronectin fragment-coated 6-well plates and add 2.5 mL of filtered retrovirus-containing supernatant or to each well (use complete TCM for mock transfection). Label each well as to the addition of retrovirus or mock media.
  5. Centrifuge the plates at 1200 x g for 30 min at room temperature.
  6. Whilst plates are spinning, collect activated T cells and count using a hemocytometer.
    1. Transduction is carried out with 5 x 10^6 ^activated splenocytes in a total of 5 mL/well. Pellet the required number of splenocytes for mock/transduction in separate tubes by centrifugation at 500 x g for 5 min.
    2. Re-suspend splenocytes at a density of 5 x 10^6 ^cells per 2.5 mL of filtered retrovirus-containing supernatant from step 2.6.4 or TCM as a negative control. Add recombinant human IL-2 (hIL-2) and recombinant mouse IL-7 (mIL-7) to a final concentration of 200 IU/mL and 4 ng/mL respectively.
  7. Collect 6-well plates from the centrifuge at the completion of step 2.6.5 and add 2.5 mL/well re-suspended splenocytes into appropriate wells to make a final volume of 5 mL/well and a final concentration of 100 U/mL hIL-2 and 2 ng/mL mIL-7.
  8. Centrifuge the plates at 1200 x g for 90 min at room temperature. After centrifugation, incubate the plates at 37 °C, 5% CO_2_ overnight.
7. **Day 5: Round 2 of transduction**
  1. Collect the recombinant human fibronectin fragment from the plates as this can be re-used. Repeat steps 2.6.2 - 2.6.5.
  2. Whilst plates are spinning, collect cells from the 1^st^ round of transduction using a Pasteur pipette. Rinse each well with 2 mL PBS, swirl and collect any remaining cells in each well. **NOTE:** Pipette up and down to re-suspend sedimented cells. Collect each control/transduction group in separate tubes.
  3. Centrifuge tubes at 500 x g for 5 min. Re-suspend cells in 2.5 mL per well of transduction with 200 IU/mL IL-2 and 4 ng/mL IL-7. Repeat steps 2.6.7 - 2.6.8.
  4. Remove cells from the centrifuge and incubate at 37 °C, 5% CO_2 _for 4 h. Collect transduced cells as in steps 2.7.2-2.7.3.
  5. Count cells, centrifuge at 500 x g for 5 min and re-suspend in complete TCM at a density of 1 x 10^6^ cells/mL with 100 U/mL hIL-2 and 2ng/mL mIL-7. Transfer to a suitably sized culture flask and incubate at 37 °C, 5% CO_2_.
  6. Add fresh TCM media containing 100U/mL hIL-2 and 2ng/mL mIL-7 every 2 days, maintaining a cell density of 1 x 10^6 ^cells/mL. **NOTE:** Harvested splenocytes contain a variety of cell types. Under these culture conditions, non T cells die off over the course of 2-3 days. After ~ 4 days in cell culture, the number of T cell is typically equivalent to the total number of harvested splenocytes on day 0.

### 3. Measurement of Transduction efficiency

1. On day 4 post transduction, collect a sample of transduced or non-transduced T cells (approximately 3 x 10^5 ^cells). Centrifuge the cell suspension at 500 x g for 5 min, discard the supernatant, wash the pelleted cells once with PBS and centrifuge again.
2. Discard the supernatant and add 100 μL of PBS containing a suitable amine reactive dye (*e.g.*, live/dead stain, 1 in 100 dilution) per well. Incubate for 15 min at room temperature in the dark.
3. Wash twice with PBS and centrifuge at 500 x g for 5 min. Discard the supernatant and incubate with 50 μL of FACS buffer containing anti-mouse CD16/CD32 antibodies for Fc receptor blocking (1 in 100 dilution). Incubate for 10 min at 4 °C.
4. Directly add 50 μL of antibody staining master mix containing anti-mouse CD4-BV786 and CD8-BV711 antibodies (final concentration of 1 μL/well in FACS buffer). Incubate for 30 min at 4 °C in dark. Repeat the wash step 3.3. Re-suspend the cells in 1% PFA buffer and keep the in the dark at 4 °C until analysis by flow cytometry.
5. Analyze cells with equivalent suitable cytometer using BV711, BV785 and mCherry fluorescence as markers of CD4 and CD8 subset and CAR expression respectively gating as in (Figure 2).

### 4. *In vitro* Validation of CAR T cell Activity

1. Seed syngeneic target CD19^+^ tumor cells with or without luciferase expression at a density of 1 x 10^4^ cells in 100 μL TCM/well in a 96-well U-bottom tissue culture plate.
2. Add 1 x 10^4^ CD19 CAR T cells/well in a volume of 100 μL/well to achieve an effector to target (E:T) ratio of 1:1. **NOTE: **E:T ratios should be established for each CAR construct and target cell line.
3. Use T cells alone and tumor cells alone as negative controls and T cells stimulated by phorbol-myristate-acetate (PMA) (50 ng/mL) and ionomycin (1 μg/mL) as positive control for Interferon gamma (IFNγ) release. Co-culture cells at 37 °C, 5% CO_2_ for 16-24 h.
4. Following co-culture, centrifuge the plates at 500 x g for 5 min and collect the supernatant for further IFNγ and IL-12p70 ELISA analysis. **NOTE: **This can be stored at -80 °C.
5. Re-suspend cell pellets in 100 μL of PBS containing luciferin (final concentration of 1.5 mg/mL). Incubate the plates for 10 min at 37 °C. Then measure the luminescence from each well with a suitable luminometer. **NOTE:** Exposure times must be optimized for cell lines and density. Representative results are shown in Figure 3a. *Ex-vivo* cytotoxicity of CAR T cells can be modified to express luciferin by co-culture with cell lines expressing target antigen. As CAR T cells kill target cells, luciferin is released, therefore a reduction in luminometry signal is correlated with cell kill. Non-transduced cells can often have an effect on target cell viability, particularly over long incubation periods. Measure the concentration of murine IFNγ and IL-12p70 in the supernatant according to the manufacturer's ELISA protocols. Representative results are shown in (Figure 3b and **3c**). *Ex-vivo* activation of CAR T cells by co-culture with cell lines expressing target antigen can be assayed by analyzing supernatant contents using ELISA. The ratio of CAR T cell to target cells and length of co-culture period must be optimized for each CAR construct, target cell line and analyte. PMA and ionomycin treatment can be used as a positive control to confirm quality of T cells and their ability to respond.

### 5. Assess Anti-cancer Activity in Mice

1. **Protocol 1**
  1. Perform 100 mg/kg intravenous (IV) delivery of cyclophosphamide into 6 to 8-week BALB/c mice. This allows tumor engraftment without significant lymphodepletion^17^ (Figure 4). **NOTE:** Establishing A20 lymphoma can take over 2 months with a suboptimal take rate. This can be improved by the use of cyclophosphamide 1 day prior to the delivery of lymphoma cells. In order to study lymphoreplete mice, we identified a dose of cyclophosphamide that could increase efficiency of lymphoma without causing lymphodepletion.
  2. The next day, inject 100 µL of 5 x 10^5^ syngeneic A20 B-cell lymphoma cells modified to express luciferase and green fluorescent protein (GFP) into mice by intravenous (IV) injection.
  3. Allow the mice to develop systemic lymphoma for ~ 17 days.
  4. Confirm the presence of systemic lymphoma by intraperitoneal (IP) injection of 100 μL of 30 mg/mL luciferin and imaging using an *in vivo* bioluminescence imaging system.
    1. Use separators to avoid signal spillover into adjacent mice. Expose mice for 1 min on the ventral side with a constant sized region of interest.
    2. Display relative light units (RLU) as photons per second (p/s). Settings must be optimized for each tumor model; use an exposure that can pick up early detection of tumors but does not lead to saturation as tumors reach endpoints.
    3. Record total RLU for each mouse with a constant sized region of interest. (Figure 5a and **b**).
  5. Inject a single dose of 1 x 10^6^ CAR T cells by IV injection into lymphoreplete mice bearing established lymphoma. **NOTE: (Important)** Dosing levels must be established for each CAR construct using a dose escalation schedule to ensure that any possible toxicities arising from CAR T cells are characterized and can be addressed. Though anti-mouse CD19 CAR T cells do not display toxicities, CAR T cells can give rise to unexpected toxicities. Where multiple CAR constructs and transduction efficiencies are not identical, the total number of T cells administered should be kept equal by the addition of non-transduced T cells into cell preparations.
  6. Monitor disease progression weekly through IP injection of 100 μL of 30 mg/mL luciferin and imaging using an *in vivo* bioluminescence imaging system (Figure 5c).
  7. Closely monitor mice for signs of toxicity and euthanize any mice that show early signs of hind limb paralysis (HLP) or pathological tumor burden before any suffering can arise. **NOTE: **Toxicities from A20 lymphoma can include hind limb paralysis through tumor invasion of the meninges. Check regularly for early signs of altered gait. Likewise, large IP tumors can arise which can lead to discomfort shown by altered behavior.
  8. Monitor survival of mice for 60 - 100 days (Figure 5d). Perform euthanasia by a schedule-1 method upon conclusion of the experiment.
2. **Protocol 2 **
  1. Deliver 200 mg/kg cyclophosphamide to 6 to 8-week old BALB/c mice by tail vein injection in 100 μL of PBS per mouse.
  2. On the following day, inject of 5 x 10^5^ syngeneic A20 B-cell lymphoma cells expressing luciferase and GFP in 100 μL PBS *via* tail vein injection.
  3. Allow the mice to develop systemic lymphomas for ~ 7-14 days
  4. Confirm systemic lymphoma by IP injection of 100 μL of 30 mg/mL luciferin and imaging using an *in vivo* bioluminescence imaging system.
  5. Perform 5 Gy total body irradiation (TBI) at 0.02 Gy/min for lymphodepletion. **NOTE:** Patients undergoing CAR T-cell treatments undergo a range of regimens to achieve lymphodepletion before the administration of CAR T cells which significantly increases the engraftment of adoptively transferred CAR T cells. This can be replicated in mice with total body irradiation (TBI) (Figure 6).
  6. On the next day, inject 1 x 10^6^ CAR T cells in 100 μL of PBS *via* tail vein injection into mice bearing established tumors.
  7. Collect blood samples *via* tail vein bleeds after 7 days.
  8. Add red cell lysis buffer to each blood sample, then prepare for flow cytometry as described in section 3. Analyze CAR T cell persistence in the circulation by flow cytometry (Figure 2). **NOTE:** Addition of counting beads immediately prior to cytometry allows determination of the number of CAR T cells per milliliter of blood.
  9. Monitor disease progress as described in steps 5.1.5 - 5.1.8 (Figure 7).

## Representative Results

For high efficiency transduction of T cells, it is necessary to obtain fresh retroviral particles. Transfection of the Plat-E cell line with pCL-Eco producer plasmid and pMP71 retrovirus plasmid gives rise to the secretion of retroviral particles into the cell supernatant. When a fluorescent marker gene, such as mCherry, is encoded in the retrovirus, successful transfection can be confirmed by fluorescence microscopy (Figure 1). Virus-containing supernatant from transfected Plat-E cells is used to transduce T cells *via* 2 rounds of spin-fection on fibronectin fragment-coated plates. The efficiency of transduction can be determined 4 days post transduction *via* flow cytometry. Successfully transduced cells express the marker gene encoded in the retrovirus (Figure 2). Transduction efficiencies range from ~ 50 - 90% efficiency with first generation receptors to ~ 10 - 40% with CAR constructs close to the retroviral packaging capacity. While marker gene expression shows successful retroviral transduction, it is paramount to show functionality of CAR T cells upon engaging with cells that express target antigen on their surface. Target cell lines modified to express luciferase can be used in luciferase assays to test the degree of cell-kill by CAR T cells directly (Figure 3A). The release of effector cytokines from CAR T cells upon co-culture with target cells, determined by ELISA, can also be used as an indirect measure of CAR T cell cytotoxicity (Figure 3B and **3C**).

CAR T cells produced in this protocol can be evaluated in lymphoreplete mice by establishing systemic A20 lymphoma with a 100 mg/kg dose of cyclophosphamide (injected intravenously), 1 day prior to IV injection of 5 x 10^5^ A20 cells (Figure 4). IP injection with luciferin and image capture using an *in vivo* bioluminescence imager can be used to monitor tumor burden using a constant ROI and exposure time throughout (Figure 5A-**C**). CAR T cells modified to express IL-12 are capable of eradicating systemic lymphoma with lymphodepleting pre-conditioning giving disease-free survival in about 25% of mice (Figure 5D). Lymphodepleting preconditioning, achieved by 5 Gy TBI 1 day prior to the IV administration of CAR T cells, significantly improves engraftment (Figure 6). In this model, first generation CAR T cells are capable of eradicating systemic A20 lymphoma, typically inducing disease-free survival in 100% of mice (Figure 7).

**Figure:**
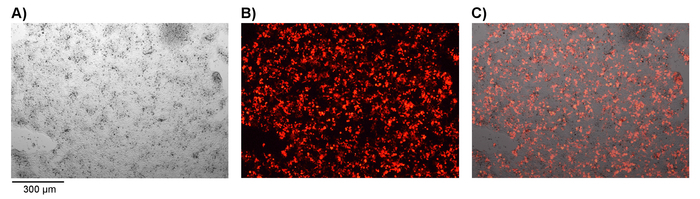

**Figure 1. Confirmation of successful transfection of Plat E cells.** Plat-E cells transfected with retroviral CAR construct and pMP71 and pcl-Eco packaging vector plasmid DNA. Successful transfection is shown by expression of the mCherry fluorescent marker gene. **A)** Bright field microscopy, **B)** fluorescence microscopy and **C)** merged images are shown. Magnification = 50X. Please click here to view a larger version of this figure.

**Figure:**
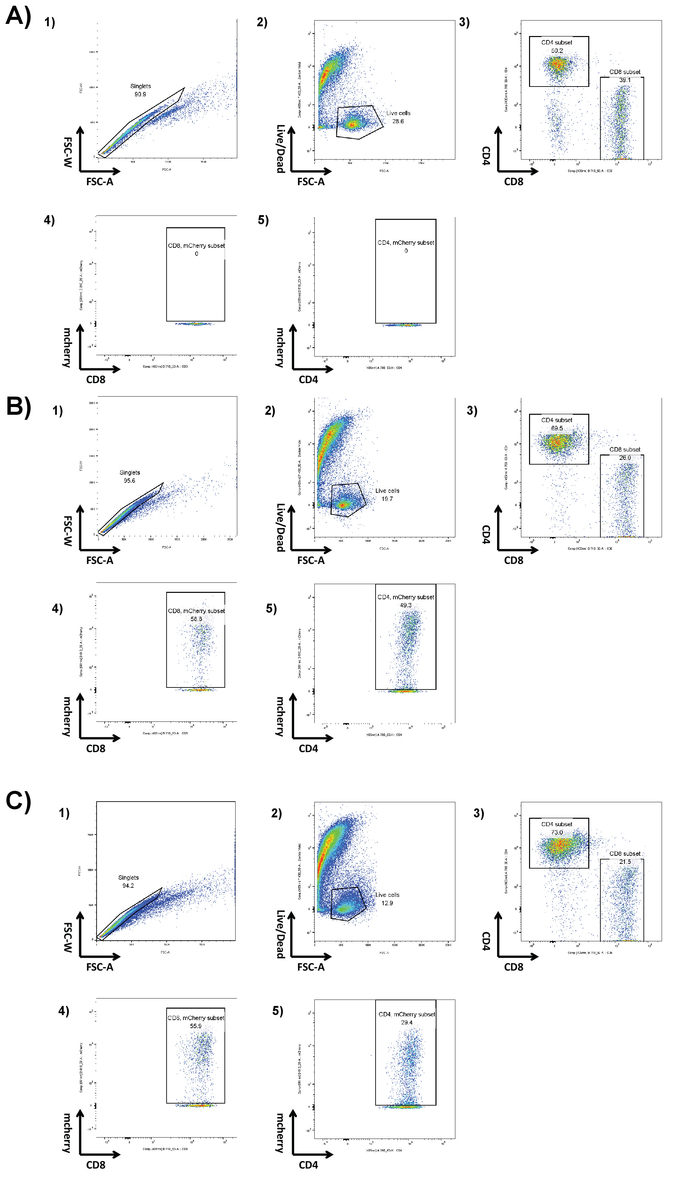

**Figure 2. Determining transduction efficiency by flow cytometry.** Flow cytometry is used to determine the transduction efficiency of the mouse T cells on day 4 post transduction, using Zombie UV live/dead, mCherry, BV711 and BV785 for the detection of the live, CAR construct, CD4 and CD8 cells, respectively. Representative results of **A)** Non-transduced, **B)** mCherry.αmCD19.mCD3z and **C)** mCherry.αmCD19.mCD3z.mIL12 are shown with gating of 1) Singlets 2) Live cells 3) CD4 and CD8 4) and 5) Assessment of mCherry positive cells expressing CAR. Please click here to view a larger version of this figure.

**Figure:**
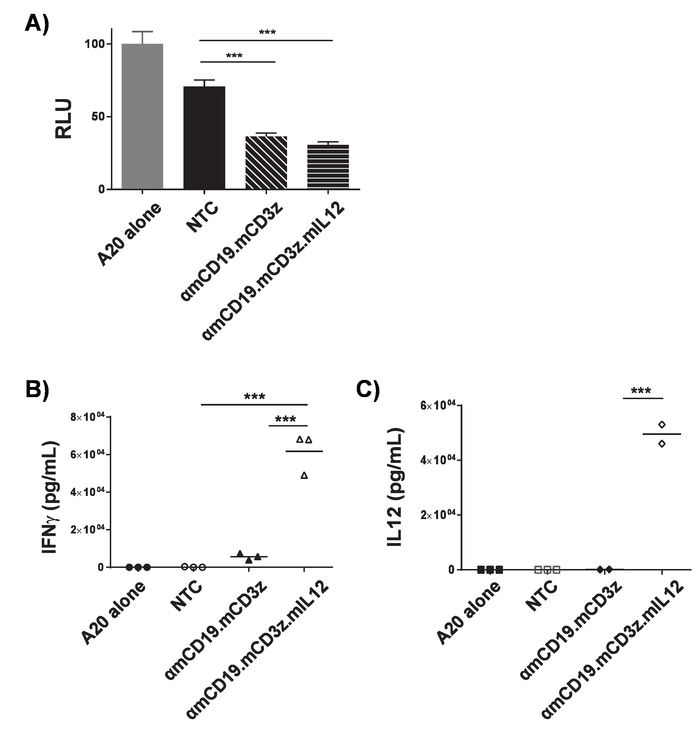

**Figure 3. Validation of CAR T-cell activity.** αmCD19 CAR T cells were co-cultured with A20 lymphoma cells modified to express luciferase (1 x 10^4^:1 x 10^4^) for 16 h in a U-bottom 96-well plate. After co-culture, cells were pelleted, and supernatant was collected. **A)** Cells were re-suspended in PBS and luminometry was used to assess the viability of the target cells. Supernatant from co-culture was assessed for the presence of IFNγ (**B**) and IL-12 (**C**). The ratio of CAR T cell to target cells and length of co-culture period must be optimized for each CAR construct and target cell line. PMA and ionomycin treatment can be used as a positive control to confirm quality of T cells and their ability cells to respond. Error bars show SD. Statistical analysis was performed using one-way ANOVA. *** *p* < 0.001). This figure has been modified from^17^. Please click here to view a larger version of this figure.

**Figure:**
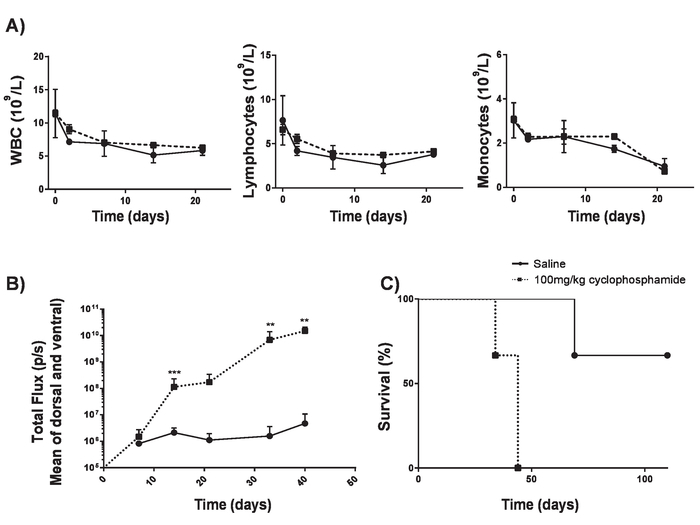

**Figure 4. Establishing A20 lymphoma without lymphodepletion.** Cyclophosphamide can increase efficiency of lymphoma induction without causing lymphodepletion. **A)** Blood counts of 6-8-week-old BALB/c mice after IV delivery of 100 mg/kg of cyclophosphamide. Error bars show SD **B)** Lymphoma burden of 6-8-week-old BALB/c mice after IV delivery of 100 mg/kg of cyclophosphamide or saline on day -1 and IV delivery of 5 x 10^5^ A20 cells on day 0 measured using a luminometer. **C**) Survival of mice in **B)**. Error bars show SD. Statistical analysis was performed using 2-way ANOVA. ** *p* < 0.01, *** *p* < 0.001). This figure has been modified from Kueberuwa *et al.*^17^. Please click here to view a larger version of this figure.

**Figure:**
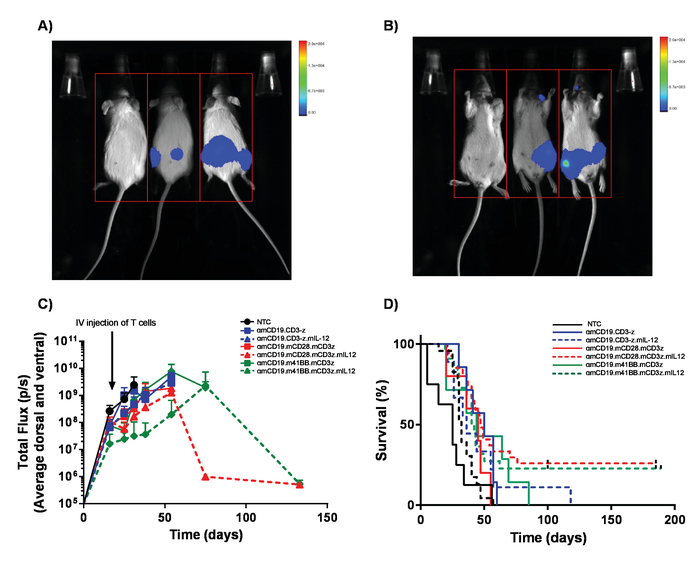

**Figure 5. Monitoring lymphoma burden and survival.** Mice bearing A20 lymphoma expressing luciferase receive 100 µL intraperitoneal (IP) injections of 30 mg/mL luciferin and were imaged using an *in vivo* bioluminescence imaging system. **A)** Mice were exposed for 1 min on the ventral side and immediately flipped over to image dorsal to pick up tumor masses on both sides of the bodies **(B)**. **C)** Representative results of the lymphoma burden of BALB/c mice receiving varying αmCD19 CAR T cells without lymphodepletion. Error bars show SEM. **D)** Survival rate of the same mice. This figure has been modified from Kueberuwa *et al.*^17^. Please click here to view a larger version of this figure.

**Figure:**
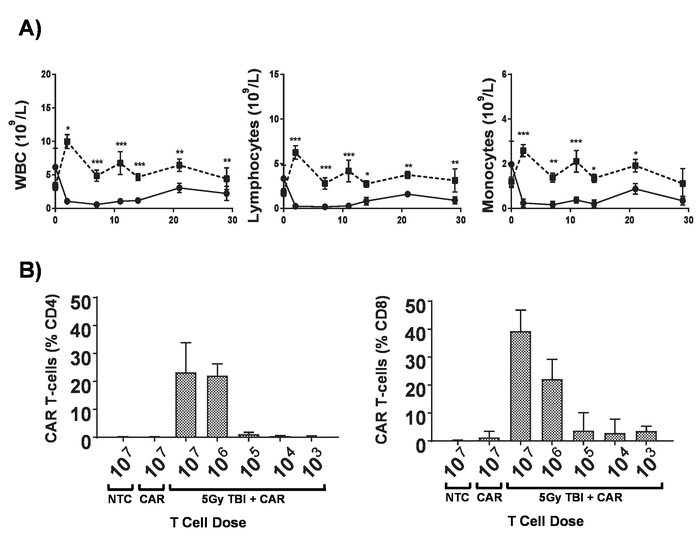

**Figure 6. Effects of lymphodepletion. A)** Blood counts of 6-8-week-old BALB/c mice after receiving 5 Gy TBI at a dose rate of 0.02 Gy/min; error bars show SD. Statistical analysis by two-way ANOVA. * *p *< 0.05, ** *p *<0.01, *** *p* < 0.001. **B)** Monitoring of CD4^+^ and CD8^+^ CAR T cells in the peripheral blood of mice by flow cytometry for the mCherry marker gene 7 days post administration. Error bars show SD. This figure has been modified from Kueberuwa *et al.*^17^. Please click here to view a larger version of this figure.

**Figure:**
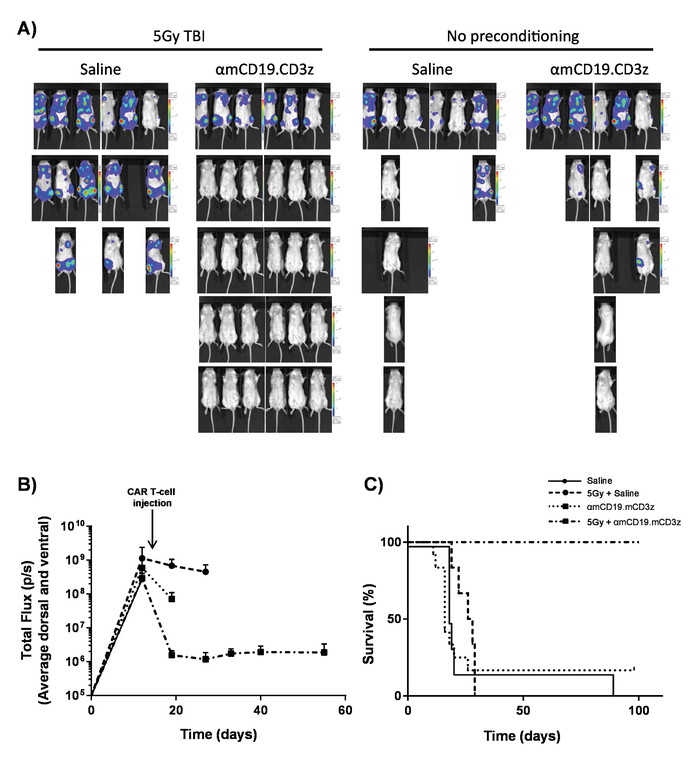

**Figure 7. CAR T cell activity with lymphodepleting pre-conditioning.** Typical results showing the effect of 5 Gy TBI the day prior to CAR T-cell administration. **A)** Imaging and **(B)** graphical displays of imaging of mice after 100 µL intraperitoneal (IP) injections of 30 mg/mL luciferin using an *in vivo* bioluminescence imaging system. Error bars show SEM. **C) **Survival of the same mice. This figure has been modified fromKueberuwa *et al.*^17^. Please click here to view a larger version of this figure.

## Discussion

Syngeneic mouse models allow the testing of disease progression and therapy while maintaining an intact immune system. This is paramount when it comes to therapies that interact with the immune system and in particular for immunotherapeutic agents.

The protocol described here has two critical work streams, the first one is genetically modifying mouse T cell to express CARs. This requires 7 days from initiation to the validation of the transduction. Concomitant with the production of CAR T cells is the establishment of systemic lymphoma in mice. Should CAR T cell production fail, or quality being insufficient, there is typically not enough time to produce replacement cells before mice succumb to lymphoma. It is therefore critical that researchers using these models accurately perform tumor dosing and disease progression studies in order to successfully time the production of CAR T cells for therapeutic administration.

Typical reasons for low T-cell transduction efficiency includes poor transfection efficiency of producer cells, typically caused by poor plasmid purity or inaccurate determination of the pH of transfection media. It is recommended to check the efficiency of producer cell transfection before proceeding with the full protocol as poor transfection will limit the efficiency of T-cell transduction. Recombinant human fibronectin fragments can be collected and stored at -20 °C for re-use, however, multiple freeze-thaws result in reduced transduction efficiency. Swift processing of mouse spleens after collection is also important for obtaining high yields of viable T cells.

It should be noted that the protocol described here utilizes A20 cells expressing luciferase. This is preferred as it provides the ability to measure systemic tumor burden by bioluminescence imaging. However, in the presence of a functional immune system, responses to luciferase could skew the results. We have previously tested immune reactions of surviving mice to marker transgenes^17^. It is key to replicate key experiments using A20 cells free of transgenes to validate that these do not play a significant role in tumor eradication by immune cells.

While clinical agents can only be used *in vivo* in immune-deficient mice, the use of mouse CAR T cells against mouse cancer cells allows us to evaluate the contributions of the immune system to therapeutic efficacy or disease progression. This protocol could be utilized for the pre-clinical evaluation of CARs targeting B-cell lymphoma or other CARs with additional modifications such as secretion of IL-12 as described here. It must be noted that although the interplay between immune cells can be evaluated in syngeneic mouse models, they may not accurately recapitulate interaction in humans *in vivo*. Of particular note, human and mouse CARs will vary in the structure which may have downstream consequences; optimal activation and cell culture conditions for growth of T cells are different^20^, tissue distribution of target antigen expression may vary between humans and mice and experienced toxicities may be radically different. It is therefore essential to utilize *ex vivo* and xenogeneic models to corroborate results.

In summary, the syngeneic lymphodepleted and lymphoreplete model of lymphoma recapitulate patients with and without prior chemo/radiotherapy. This provides a model system in which to mimic the clinical settings to allow the testing of a range of therapeutic strategies that will be important with the coming wave of new immune therapy agents.

With the use of pre-conditioning, it will be noted that all the mice typically clear the lymphoma. With up to 90% complete response rates in humans, this is representative. However, the challenges for CD19 CAR T-cell therapy will hinge on preventing the high frequency of relapses observed that are often CD19. Relapses have not been observed in this model up to, and often beyond 100 days. Modifications to mimic the relapses seen in the clinic could help with the future challenges of CD19 CAR T-cell therapy.

## Disclosures

David Gilham works for Celyad which is involved in the production of CAR T cells. The rest of the authors have nothing to disclose.
